# Crystal structure of *trans*-bis­(ethane-1,2-diamine-κ^2^ *N*,*N*′)bis­(thio­cyanato-κ*N*)chromium(III) perchlorate from synchrotron data

**DOI:** 10.1107/S2056989015009184

**Published:** 2015-05-20

**Authors:** Dohyun Moon, Jong-Ha Choi

**Affiliations:** a Pohang Accelerator Laboratory, POSTECH, Pohang 790-784, Republic of Korea; b Department of Chemistry, Andong National University, Andong 760-749, Republic of Korea

**Keywords:** crystal structure, synchrotron radiation, ethane-1,2-di­amine, thio­cyanate, *trans*-geometry, chromium(III) complex, hydrogen bonds

## Abstract

The structure of the title compound, [Cr(NCS)_2_(C_2_H_8_N_2_)_2_]ClO_4_, has been determined from synchroton data. The asymmetric unit consists of one half of a centrosymmetric Cr^III^ complex cation and half of a perchlorate anion with the Cl atom on a twofold rotation axis. The Cr^III^ ion is coordinated by the four N atoms of two ethane-1,2-di­amine (en) ligands in the equatorial plane and two N-bound thio­cyanate (NCS^−^) anions in a *trans*-axial arrangement, displaying a slightly distorted octa­hedral geometry with crystallographic inversion symmetry. The Cr—N(en) bond lengths are in the range 2.053 (16)–2.09 (2) Å, while the Cr—N(thio­cyanate) bond length is 1.983 (2) Å. The five-membered en rings are disordered over two sites, with occupancy ratios of 0.522 (16):0.478 (16). Each ClO_4_^−^ anion is disordered over two sites with equal occupancy. The crystal structure is stabilized by inter­molecular hydrogen bonds involving the en NH_2_ groups as donors and perchlorate O and thio­cyanate S atoms as acceptors.

## Abstract 2

The centrosymmetric Cr^III^ ion in the title compound shows a distorted octa­hedral coordination with four N atoms of two ethane-1,2-di­amine ligands in the equatorial plane and two N-coordinated NCS^−^ groups in *trans*-axial positions. The ethane-1,2-di­amine ligand in the complex cation and the ClO_4_^−^ anion are both disordered.

## Chemical context   

Considerable attention has been focussed for some time on metal complexes containing thio­cyanate ligands because of their ability to coordinate through either the N or S atoms. Ethane-1,2-di­amine (en) can coordinate to a central metal ion as a bidentate ligand *via* the two N atoms, forming a five-membered chelate ring. The [Cr(NCS)_2_(en)_2_]^+^ cation can form either *trans* or *cis* geometric isomers. *Trans* and *cis* isomers of the complex cation with SCN^−^ or ClO_4_^−^ counter-anions have been prepared and their IR spectral properties reported (House, 1973 ▸; Sandrini *et al.*, 1978 ▸; De *et al.*, 1987 ▸). IR and electronic spectral properties are useful in determining the geometric isomers of chromium(III) complexes with mixed ligands (Choi, 2000 ▸; Choi *et al.*, 2004 ▸; Choi & Moon, 2014 ▸). However, it should be noted that the geometric assignments based on spectroscopic studies are not always definitive.
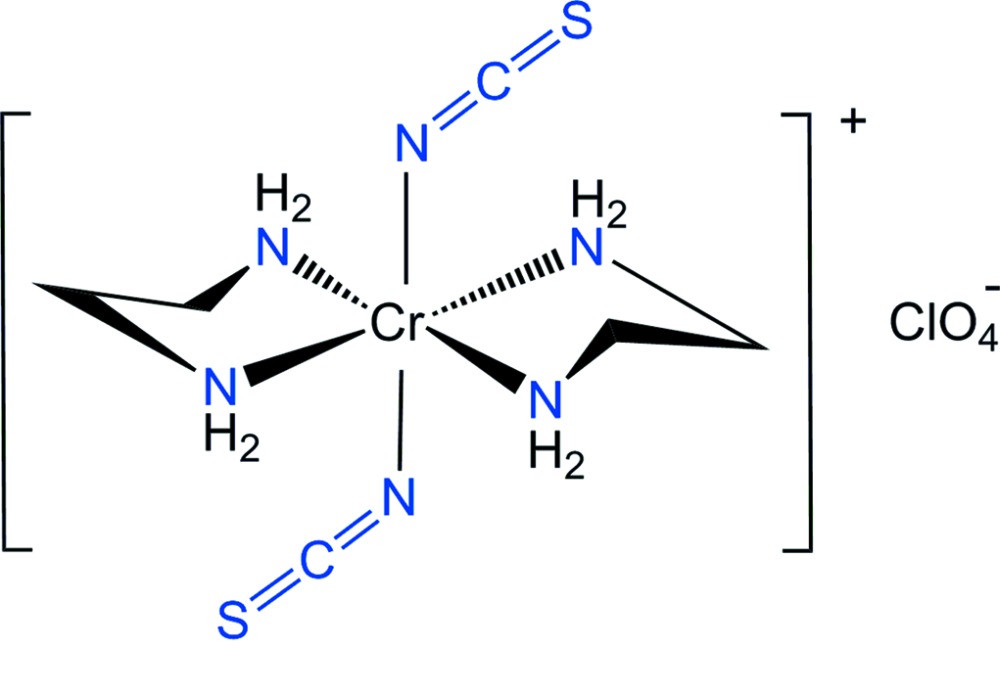


In a recent publication, we described the synthesis and crystal structure of *trans*-[Cr(NCS)_2_(en)_2_]_2_[ZnCl_4_] (Moon & Choi, 2015 ▸). The asymmetric unit of this complex contained four halves of centrosymmetric [Cr(NCS)_2_(en)_2_]^+^ complex cations and one [ZnCl_4_]^2−^ anion. To compare and contrast this structure with a complex of this cation with a different counter-anion we report here the structure of *trans*-[Cr(NCS)_2_(en)_2_]ClO_4_, (I).

## Structural commentary   

**Figure 1:**
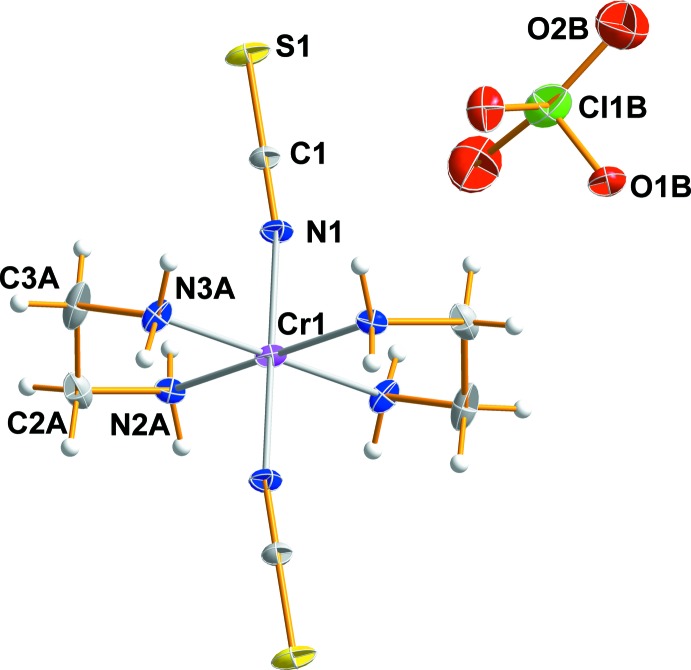
The mol­ecular structure of (I), drawn with 20% probability displacement ellipsoids. Atoms of the minor disorder components have been omitted for clarity.

Fig. 1 ▸ shows an ellipsoid plot of *trans*-[Cr(NCS)_2_(en)_2_]ClO_4_, (I), with the atom-numbering scheme. In the structure of (I), there is a centrosymmetric Cr^III^ complex cation with two en ligands bound through their N atoms in equatorial sites and the two axial N-bound thio­cyanate anions in a *trans* configuration. The asymmetric unit is composed of half of one complex cation and half a ClO_4_^−^ anion. The Cr^III^ atom is located on a crystallographic centre of symmetry, so this complex cation has mol­ecular *C_i_* symmetry, while the the Cl atom of the perchlorate anion lies on a twofold rotation axis. The bidentate en ligand adopts a stable *gauche* conformation similar to that observed in related compounds (Brenčič & Leban, 1981 ▸; Choi *et al.*, 2010 ▸). The Cr—N bond lengths for the en ligand range from 2.053 (16) to 2.09 (2) Å, and these bond lengths are in good agreement with those observed in *trans*-[CrF_2_(en)_2_]ClO_4_ (Brenčič & Leban, 1981 ▸), *trans*-[CrBr_2_(en)_2_]ClO_4_ (Choi *et al.*, 2010 ▸), *trans*-[CrCl_2_(Me_2_tn)_2_]_2_ZnCl_4_ (Me_2_tn = 2,2-di­methyl­propane-1,3-di­amine; Choi *et al.*, 2011 ▸) and *trans-*[CrF_2_(2,2,3-tet)]ClO_4_ (2,2,3-tet = 1,4,7,11-tetra­aza­undecane; Choi & Moon, 2014 ▸). The Cr—N(thio­cyanate) bond length is 1.983 (2) Å and is similar to the average values of 1.985 (2), 1.995 (6), 1.983 (2) and 1.996 (15) Å found in *trans*-[Cr(NCS)_2_(en)_2_]_2_ZnCl_4_ (Moon & Choi, 2015 ▸), *trans*-[Cr(NCS)_2_(cyclam)]_2_ZnCl_4_ (cyclam = 1,4,8,11-tetra­aza­cyclo­tetra­decane (Moon *et al.*, 2015 ▸), *trans*-[Cr(NCS)_2_(Me_2_tn)_2_]NCS (Choi & Lee, 2009 ▸) and *cis*-[Cr(NCS)_2_(cyclam)]NCS (Moon *et al.*, 2013 ▸), respectively. The N-coordinated iso­thio­cyanate group is almost linear, with an N—C—S angle of 179.3 (3)°. The ClO_4_^−^ counter-anion lies well outside the coordination sphere of the complex and, because of significant disorder, the tetra­hedral geometry of this anion is severely distorted.

## Supra­molecular features   

**Figure 2:**
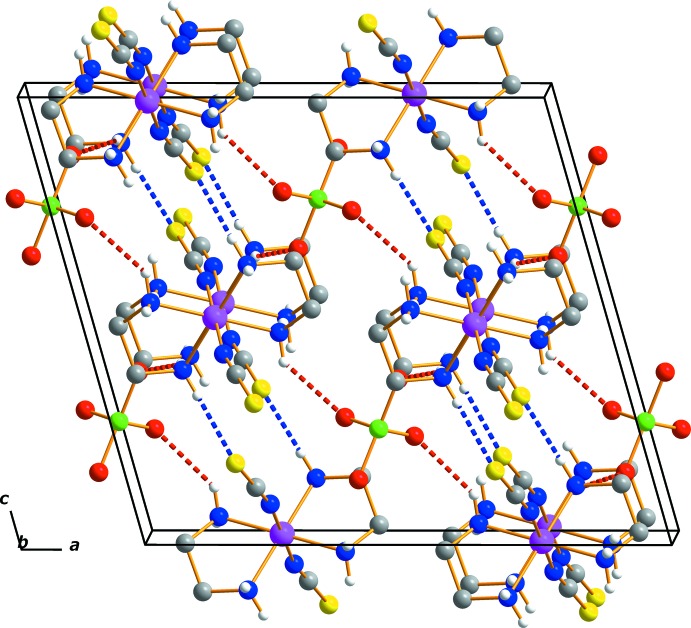
The crystal packing of (I), viewed perpendicular to the *ac* plane. Dashed lines represent N—H⋯O (red) and N—H⋯S (blue) hydrogen-bonding inter­actions, respectively. The minor disorder components and C-bound H atoms have been omitted for clarity.

In the crystal, an N—H⋯S hydrogen bond links neighbouring cations, while a series of N—H⋯O contacts link the cations to neighbouring anions (Table 1 ▸). An extensive array of these contacts generate a three-dimensional network of mol­ecules stacked along the *b*-axis direction (Fig. 2 ▸). These hydrogen-bonded networks help to stabilize the crystal structure.

## Database survey   

A search of the Cambridge Structural Database (Version 5.36, last update February 2015; Groom & Allen, 2014 ▸) indicates a total of 13 hits for Cr^III^ complexes with a [Cr*L*_2_(en)_2_]^+^ unit. The crystal structures of *trans*-[CrCl_2_(en)_2_]Cl·HCl·2H_2_O (Ooi *et al.*, 1960 ▸), *trans*-[CrF_2_(en)_2_]*X* (*X* = ClO_4_, Cl, Br) (Brenčič & Leban, 1981 ▸), *cis*-[CrF_2_(en)_2_]ClO_4_ (Brenčič *et al.*, 1987 ▸), *trans*-[CrBr_2_(en)_2_]ClO_4_ (Choi *et al.*, 2010 ▸) have been reported previously. Recently, we have also reported the closely related crystal structure of [Cr(NCS)_2_(en)_2_]_2_[ZnCl_4_], in which there are four crystallographically independent Cr^III^ complex cations that also adopt a *trans* configuration. However, a crystal structure of [Cr(NCS)_2_(en)_2_]^+^ with a ClO_4_ anion has not been reported previously.

## Synthesis and crystallization   

All chemicals were reagent grade materials and were used without further purification. The title compound, *trans*-[Cr(NCS)_2_(en)_2_]ClO_4_ was prepared according to the literature method (Sandrini *et al.*, 1978 ▸). The crude perchlorate salt (0.33 g) was dissolved in 20 mL of 0.1 *M* HCl at 333 K. The filtrate was added to 6 mL of 60% HClO_4_. The resulting solution was allowed to stand at room temperature for 2 d to give orange block-like crystals suitable for X-ray structural analysis. IR spectrum (KBr, cm^−1^) : 3247 (*vs*), 3208 (*vs*), 3131 (*vs*) and 3097 (*vs*) (ν NH), 2966 (*s*), 2955 (*s*) and 2893 (*s*) (ν CH), 2077 (*vs*) (ν_*a*_ CN), 1586 (*vs*) (δ NH_2_), 1459 (*s*) (δ CH_2_), 1365 (*m*) (ν CN), 1326 (*s*) (ω NH_2_), 1290 (*vs*) (ω CH_2_), 1146 (*vs*) (γ NH_2_), 1117 (*vs*) (ν CN), 1088 (*vs*) (ν_*a*_ Cl—O), 1047 (*vs*) (γ CH_2_), 1007 (*s*), 983 (*s*), 873 (*m*) (ρ CH_2_), 849 (*w*) (ρ NH_2_), 729 (*vs*), 636 (*s*) and 626 (*vs*) (δ OClO), 558 (*vs*), 559 (*s*) (δ CCC), 501 (*vs*), 478 (*s*) (δ NCS), 444 (*m*) and 419 (*m*) (ν Cr—N).

## Refinement   

Crystal data, data collection and structure refinement details are summarized in Table 2 ▸. In the title compound, the ethane-1,2-di­amine group is disordered with atoms N2*A*/N2*B*, C2*A*/C2*B*, C3*A*/C3*B* and N3*A*/N3*B* positionally disordered over two sets of sites with a refined occupancy ratio of 0.522 (16):0.478 (16). The half mol­ecules of each distorted perchlorate anion are disordered over two sites of equal occupancy, with atoms Cl1*B*/Cl1*C* and O2*B*/O1*C* refined using EXYZ/EADP constraints. All H atoms were placed in geometrically idealized positions and constrained to ride on their parent atoms, with C—H = 0.97 Å and N—H = 0.89 Å, and with *U*_iso_(H) values of 1.2 of the parent atoms.

## Supplementary Material

Crystal structure: contains datablock(s) I. DOI: 10.1107/S2056989015009184/sj5459sup1.cif

Structure factors: contains datablock(s) I. DOI: 10.1107/S2056989015009184/sj5459Isup2.hkl

CCDC reference: 1400767

Additional supporting information:  crystallographic information; 3D view; checkCIF report

## Figures and Tables

**Table 1:** Hydrogen-bond geometry (, )

*D*H*A*	*D*H	H*A*	*D* *A*	*D*H*A*
N2*A*H2*A*1S1^i^	0.89	2.45	3.324(17)	167
N2*A*H2*A*2O2*B* ^ii^	0.89	2.41	3.187(19)	146
N3*A*H3*A*1O1*B* ^iii^	0.89	2.58	3.282(16)	136
N2*B*H2*B*1S1^i^	0.89	2.77	3.459(17)	135
N3*B*H3*B*1O2*C* ^iii^	0.89	2.45	3.22(2)	145
N3*B*H3*B*2S1^iv^	0.89	2.38	3.255(18)	166

Symmetry codes: (i) 
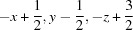
; (ii) 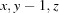; (iii) 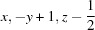; (iv) 
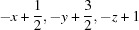
.

**Table 2:** Experimental details

Crystal data	
Chemical formula	[Cr(NCS)_2_(C_2_H_8_N_2_)_2_]ClO_4_
*M*_r_	387.82
Crystal system, space group	Monoclinic, *C*2/*c*
Temperature (K)	260
*a*, *b*, *c* ()	15.599(3), 7.4440(15), 13.792(3)
()	105.83(3)
*V* (^3^)	1540.8(6)
*Z*	4
Radiation type	Synchrotron, = 0.630
(mm^1^)	0.86
Crystal size (mm)	0.14 0.13 0.13

Data collection	
Diffractometer	ADSC Q210 CCD area detector
Absorption correction	Empirical (using intensity measurements) (*HKL3000sm SCALEAPCK*; Otwinowski Minor, 1997 ▸)
*T*_min_, *T*_max_	0.893, 0.897
No. of measured, independent and observed [*I* > 2(*I*)] reflections	8172, 2121, 2019
*R*_int_	0.015
(sin /)_max_ (^1^)	0.696

Refinement	
*R*[*F* ^2^ > 2(*F* ^2^)], *wR*(*F* ^2^), *S*	0.060, 0.178, 1.09
No. of reflections	2121
No. of parameters	140
H-atom treatment	H-atom parameters constrained
_max_, _min_ (e ^3^)	0.74, 1.12

Computer programs: *PAL ADSC Quantum-210 ADX Program* (Arvai Nielsen, 1983 ▸), *HKL3000sm* (Otwinowski Minor, 1997 ▸), *SHELXT2014/5* (Sheldrick, 2015*a* ▸), *SHELXL2014/7* (Sheldrick, 2015*b* ▸), *DIAMOND* (Putz Brandenburg, 2014 ▸) and *publCIF* (Westrip, 2010 ▸).

## supplementary crystallographic information

### Crystal data

**Table:**

[Cr(NCS)_2_(C_2_H_8_N_2_)_2_]ClO_4_	*F*(000) = 796
*M**_r_* = 387.82	*D*_x_ = 1.672 Mg m^−^^3^
Monoclinic, *C*2/*c*	Synchrotron radiation, λ = 0.630 Å
*a* = 15.599 (3) Å	Cell parameters from 46962 reflections
*b* = 7.4440 (15) Å	θ = 0.4–33.6°
*c* = 13.792 (3) Å	µ = 0.86 mm^−^^1^
β = 105.83 (3)°	*T* = 260 K
*V* = 1540.8 (6) Å^3^	Block, orange
*Z* = 4	0.14 × 0.13 × 0.13 mm

### Data collection

**Table:**

ADSC Q210 CCD area-detector diffractometer	2019 reflections with *I* > 2σ(*I*)
Radiation source: PLSII 2D bending magnet	*R*_int_ = 0.015
ω scan	θ_max_ = 26.0°, θ_min_ = 2.7°
Absorption correction: empirical (using intensity measurements) (*HKL3000sm SCALEAPCK*; Otwinowski & Minor, 1997)	*h* = −21→21
*T*_min_ = 0.893, *T*_max_ = 0.897	*k* = −10→10
8172 measured reflections	*l* = −19→19
2121 independent reflections	

### Refinement

**Table:**

Refinement on *F*^2^	Hydrogen site location: inferred from neighbouring sites
Least-squares matrix: full	H-atom parameters constrained
*R*[*F*^2^ > 2σ(*F*^2^)] = 0.060	*w* = 1/[σ^2^(*F*_o_^2^) + (0.1146*P*)^2^ + 2.4721*P*] where *P* = (*F*_o_^2^ + 2*F*_c_^2^)/3
*wR*(*F*^2^) = 0.178	(Δ/σ)_max_ < 0.001
*S* = 1.09	Δρ_max_ = 0.74 e Å^−^^3^
2121 reflections	Δρ_min_ = −1.12 e Å^−^^3^
140 parameters	Extinction correction: *SHELXL2014*/7 (Sheldrick, 2015*b*), Fc^*^=kFc[1+0.001xFc^2^λ^3^/sin(2θ)]^-1/4^
0 restraints	Extinction coefficient: 0.045 (12)

### Special details

**Table:**

Geometry. All e.s.d.'s (except the e.s.d. in the dihedral angle between two l.s. planes) are estimated using the full covariance matrix. The cell e.s.d.'s are taken into account individually in the estimation of e.s.d.'s in distances, angles and torsion angles; correlations between e.s.d.'s in cell parameters are only used when they are defined by crystal symmetry. An approximate (isotropic) treatment of cell e.s.d.'s is used for estimating e.s.d.'s involving l.s. planes.

### Fractional atomic coordinates and isotropic or equivalent isotropic displacement parameters (Å^2^)

**Table:**

	*x*	*y*	*z*	*U*_iso_*/*U*_eq_	Occ. (<1)
Cr1	0.2500	0.2500	0.5000	0.0273 (3)	
S1	0.21080 (8)	0.77661 (11)	0.67477 (8)	0.0586 (3)	
N1	0.24831 (15)	0.4775 (3)	0.57426 (17)	0.0433 (5)	
C1	0.23290 (16)	0.6026 (3)	0.61573 (17)	0.0363 (5)	
N2A	0.3423 (12)	0.1308 (19)	0.6213 (13)	0.036 (2)	0.522 (16)
H2A1	0.3272	0.1503	0.6781	0.043*	0.522 (16)
H2A2	0.3442	0.0127	0.6118	0.043*	0.522 (16)
N3A	0.3624 (11)	0.337 (2)	0.4641 (10)	0.043 (3)	0.522 (16)
H3A1	0.3553	0.3266	0.3981	0.052*	0.522 (16)
H3A2	0.3722	0.4525	0.4807	0.052*	0.522 (16)
C2A	0.4311 (5)	0.2126 (10)	0.6277 (8)	0.057 (2)	0.522 (16)
H2A3	0.4784	0.1375	0.6678	0.068*	0.522 (16)
H2A4	0.4355	0.3305	0.6587	0.068*	0.522 (16)
C3A	0.4385 (5)	0.2274 (14)	0.5199 (10)	0.066 (3)	0.522 (16)
H3A3	0.4943	0.2842	0.5191	0.079*	0.522 (16)
H3A4	0.4362	0.1092	0.4897	0.079*	0.522 (16)
N2B	0.3570 (13)	0.164 (2)	0.6143 (14)	0.041 (3)	0.478 (16)
H2B1	0.3654	0.2382	0.6667	0.049*	0.478 (16)
H2B2	0.3464	0.0548	0.6342	0.049*	0.478 (16)
N3B	0.3502 (13)	0.341 (3)	0.4378 (9)	0.040 (2)	0.478 (16)
H3B1	0.3527	0.2732	0.3856	0.048*	0.478 (16)
H3B2	0.3396	0.4543	0.4167	0.048*	0.478 (16)
C2B	0.4369 (4)	0.1614 (15)	0.5773 (8)	0.056 (2)	0.478 (16)
H2B3	0.4355	0.0582	0.5339	0.067*	0.478 (16)
H2B4	0.4902	0.1541	0.6334	0.067*	0.478 (16)
C3B	0.4370 (5)	0.3297 (19)	0.5203 (7)	0.060 (3)	0.478 (16)
H3B3	0.4869	0.3303	0.4911	0.072*	0.478 (16)
H3B4	0.4427	0.4322	0.5651	0.072*	0.478 (16)
Cl1B	0.5000	0.7072 (3)	0.7500	0.0989 (7)	0.5
O1B	0.4393 (4)	0.5672 (9)	0.7711 (5)	0.0762 (16)	0.5
O2B	0.4350 (6)	0.7462 (8)	0.6376 (6)	0.159 (3)	0.5
Cl1C	0.5000	0.7072 (3)	0.7500	0.0989 (7)	0.5
O1C	0.4350 (6)	0.7462 (8)	0.6376 (6)	0.159 (3)	0.5
O2C	0.4488 (11)	0.8416 (15)	0.7860 (8)	0.152 (5)	0.5

### Atomic displacement parameters (Å^2^)

**Table:**

	*U*^11^	*U*^22^	*U*^33^	*U*^12^	*U*^13^	*U*^23^
Cr1	0.0366 (4)	0.0229 (3)	0.0251 (3)	0.00353 (14)	0.0128 (2)	−0.00135 (14)
S1	0.0988 (7)	0.0298 (4)	0.0630 (6)	0.0072 (3)	0.0492 (5)	−0.0070 (3)
N1	0.0553 (12)	0.0327 (11)	0.0427 (10)	0.0052 (9)	0.0145 (9)	−0.0097 (8)
C1	0.0472 (12)	0.0289 (10)	0.0356 (10)	0.0012 (9)	0.0163 (9)	−0.0012 (8)
N2A	0.050 (5)	0.024 (3)	0.034 (3)	−0.001 (2)	0.011 (3)	0.004 (2)
N3A	0.047 (5)	0.036 (3)	0.056 (7)	0.006 (3)	0.028 (5)	0.017 (5)
C2A	0.048 (3)	0.044 (3)	0.065 (5)	−0.003 (2)	−0.006 (3)	−0.001 (3)
C3A	0.038 (3)	0.044 (4)	0.121 (8)	0.008 (3)	0.031 (4)	0.026 (5)
N2B	0.048 (6)	0.044 (8)	0.031 (3)	0.012 (5)	0.014 (3)	0.005 (4)
N3B	0.050 (5)	0.043 (4)	0.031 (4)	0.005 (3)	0.019 (4)	0.002 (3)
C2B	0.041 (3)	0.073 (5)	0.051 (5)	0.017 (3)	0.007 (3)	0.003 (4)
C3B	0.045 (3)	0.068 (7)	0.070 (4)	−0.014 (4)	0.023 (3)	−0.013 (4)
Cl1B	0.1112 (15)	0.0671 (10)	0.1316 (18)	0.000	0.0553 (13)	0.000
O1B	0.074 (3)	0.073 (4)	0.083 (4)	−0.009 (3)	0.024 (3)	0.029 (3)
O2B	0.152 (6)	0.199 (8)	0.130 (5)	0.028 (4)	0.045 (5)	0.035 (4)
Cl1C	0.1112 (15)	0.0671 (10)	0.1316 (18)	0.000	0.0553 (13)	0.000
O1C	0.152 (6)	0.199 (8)	0.130 (5)	0.028 (4)	0.045 (5)	0.035 (4)
O2C	0.255 (15)	0.095 (7)	0.121 (8)	−0.038 (9)	0.076 (9)	−0.014 (6)

### Geometric parameters (Å, º)

**Table:**

Cr1—N1	1.983 (2)	C3A—H3A3	0.9700
Cr1—N1^i^	1.983 (2)	C3A—H3A4	0.9700
Cr1—N3A^i^	2.053 (16)	N2B—C2B	1.471 (18)
Cr1—N3A	2.053 (16)	N2B—H2B1	0.8900
Cr1—N2B	2.06 (2)	N2B—H2B2	0.8900
Cr1—N2B^i^	2.06 (2)	N3B—C3B	1.514 (17)
Cr1—N2A^i^	2.085 (19)	N3B—H3B1	0.8900
Cr1—N2A	2.085 (19)	N3B—H3B2	0.8900
Cr1—N3B^i^	2.09 (2)	C2B—C3B	1.479 (17)
Cr1—N3B	2.09 (2)	C2B—H2B3	0.9700
S1—C1	1.617 (3)	C2B—H2B4	0.9700
N1—C1	1.152 (3)	C3B—H3B3	0.9700
N2A—C2A	1.493 (15)	C3B—H3B4	0.9700
N2A—H2A1	0.8900	Cl1B—O1B^ii^	1.489 (6)
N2A—H2A2	0.8900	Cl1B—O1B	1.489 (6)
N3A—C3A	1.475 (16)	Cl1B—O2B^ii^	1.630 (8)
N3A—H3A1	0.8900	Cl1B—O2B	1.630 (8)
N3A—H3A2	0.8900	Cl1C—O2C^ii^	1.450 (13)
C2A—C3A	1.527 (17)	Cl1C—O2C	1.450 (13)
C2A—H2A3	0.9700	Cl1C—O1C^ii^	1.630 (8)
C2A—H2A4	0.9700	Cl1C—O1C	1.630 (8)
			
N1—Cr1—N1^i^	180.0	N2A—C2A—H2A4	110.4
N1—Cr1—N3A^i^	90.8 (5)	C3A—C2A—H2A4	110.4
N1^i^—Cr1—N3A^i^	89.2 (5)	H2A3—C2A—H2A4	108.6
N1—Cr1—N3A	89.2 (5)	N3A—C3A—C2A	106.5 (10)
N1^i^—Cr1—N3A	90.8 (5)	N3A—C3A—H3A3	110.4
N3A^i^—Cr1—N3A	180.0	C2A—C3A—H3A3	110.4
N1—Cr1—N2B	89.5 (5)	N3A—C3A—H3A4	110.4
N1^i^—Cr1—N2B	90.5 (5)	C2A—C3A—H3A4	110.4
N1—Cr1—N2B^i^	90.5 (5)	H3A3—C3A—H3A4	108.6
N1^i^—Cr1—N2B^i^	89.5 (5)	C2B—N2B—Cr1	109.0 (9)
N2B—Cr1—N2B^i^	180.0 (9)	C2B—N2B—H2B1	109.9
N1—Cr1—N2A^i^	87.0 (4)	Cr1—N2B—H2B1	109.9
N1^i^—Cr1—N2A^i^	93.0 (5)	C2B—N2B—H2B2	109.9
N3A^i^—Cr1—N2A^i^	83.1 (5)	Cr1—N2B—H2B2	109.9
N3A—Cr1—N2A^i^	96.9 (5)	H2B1—N2B—H2B2	108.3
N1—Cr1—N2A	93.0 (4)	C3B—N3B—Cr1	106.7 (8)
N1^i^—Cr1—N2A	87.0 (4)	C3B—N3B—H3B1	110.4
N3A^i^—Cr1—N2A	96.9 (5)	Cr1—N3B—H3B1	110.4
N3A—Cr1—N2A	83.1 (5)	C3B—N3B—H3B2	110.4
N2A^i^—Cr1—N2A	180.0	Cr1—N3B—H3B2	110.4
N1—Cr1—N3B^i^	87.1 (5)	H3B1—N3B—H3B2	108.6
N1^i^—Cr1—N3B^i^	92.9 (5)	N2B—C2B—C3B	107.1 (10)
N2B—Cr1—N3B^i^	97.2 (5)	N2B—C2B—H2B3	110.3
N2B^i^—Cr1—N3B^i^	82.8 (5)	C3B—C2B—H2B3	110.3
N1—Cr1—N3B	92.9 (5)	N2B—C2B—H2B4	110.3
N1^i^—Cr1—N3B	87.1 (5)	C3B—C2B—H2B4	110.3
N2B—Cr1—N3B	82.8 (5)	H2B3—C2B—H2B4	108.5
N2B^i^—Cr1—N3B	97.2 (5)	C2B—C3B—N3B	108.6 (10)
N3B^i^—Cr1—N3B	180.0	C2B—C3B—H3B3	110.0
C1—N1—Cr1	168.7 (2)	N3B—C3B—H3B3	110.0
N1—C1—S1	179.3 (3)	C2B—C3B—H3B4	110.0
C2A—N2A—Cr1	107.5 (7)	N3B—C3B—H3B4	110.0
C2A—N2A—H2A1	110.2	H3B3—C3B—H3B4	108.4
Cr1—N2A—H2A1	110.2	O1B^ii^—Cl1B—O1B	91.2 (5)
C2A—N2A—H2A2	110.2	O1B^ii^—Cl1B—O2B^ii^	92.7 (4)
Cr1—N2A—H2A2	110.2	O1B—Cl1B—O2B^ii^	101.7 (3)
H2A1—N2A—H2A2	108.5	O1B^ii^—Cl1B—O2B	101.7 (3)
C3A—N3A—Cr1	108.5 (8)	O1B—Cl1B—O2B	92.7 (4)
C3A—N3A—H3A1	110.0	O2B^ii^—Cl1B—O2B	159.4 (5)
Cr1—N3A—H3A1	110.0	O2C^ii^—Cl1C—O2C	92.7 (9)
C3A—N3A—H3A2	110.0	O2C^ii^—Cl1C—O1C^ii^	86.8 (6)
Cr1—N3A—H3A2	110.0	O2C—Cl1C—O1C^ii^	79.0 (6)
H3A1—N3A—H3A2	108.4	O2C^ii^—Cl1C—O1C	79.0 (6)
N2A—C2A—C3A	106.7 (9)	O2C—Cl1C—O1C	86.8 (6)
N2A—C2A—H2A3	110.4	O1C^ii^—Cl1C—O1C	159.4 (5)
C3A—C2A—H2A3	110.4		
			
Cr1—N2A—C2A—C3A	−42.0 (11)	Cr1—N2B—C2B—C3B	44.2 (13)
Cr1—N3A—C3A—C2A	−44.8 (13)	N2B—C2B—C3B—N3B	−56.3 (15)
N2A—C2A—C3A—N3A	57.9 (14)	Cr1—N3B—C3B—C2B	40.2 (12)

Symmetry codes: (i) −*x*+1/2, −*y*+1/2, −*z*+1; (ii) −*x*+1, *y*, −*z*+3/2.

### Hydrogen-bond geometry (Å, º)

**Table:**

*D*—H···*A*	*D*—H	H···*A*	*D*···*A*	*D*—H···*A*
N2*A*—H2*A*1···S1^iii^	0.89	2.45	3.324 (17)	167
N2*A*—H2*A*2···O2*B*^iv^	0.89	2.41	3.187 (19)	146
N3*A*—H3*A*1···O1*B*^v^	0.89	2.58	3.282 (16)	136
N2*B*—H2*B*1···S1^iii^	0.89	2.77	3.459 (17)	135
N3*B*—H3*B*1···O2*C*^v^	0.89	2.45	3.22 (2)	145
N3*B*—H3*B*2···S1^vi^	0.89	2.38	3.255 (18)	166

Symmetry codes: (iii) −*x*+1/2, *y*−1/2, −*z*+3/2; (iv) *x*, *y*−1, *z*; (v) *x*, −*y*+1, *z*−1/2; (vi) −*x*+1/2, −*y*+3/2, −*z*+1.

## References

[bb1] Arvai, A. J. & Nielsen, C. (1983). *ADSC Quantum-210 ADX*. Area Detector System Corporation, Poway, CA, USA.

[bb2] Brenčič, J. V. & Leban, I. (1981). *Z. Anorg. Allg. Chem.* **480**, 213–219.

[bb3] Brenčič, J. V., Leban, I. & Polanc, I. (1987). *Acta Cryst.* C**43**, 885–887.

[bb4] Choi, J.-H. (2000). *Chem. Phys.* **256**, 29–35.

[bb5] Choi, J.-H., Clegg, W., Harrington, R. W. & Lee, S. H. (2010). *J. Chem. Crystallogr.* **40**, 567–571.

[bb6] Choi, J.-H., Joshi, T. & Spiccia, L. (2011). *Z. Anorg. Allg. Chem.* **637**, 1194–1198.

[bb7] Choi, J.-H. & Lee, S. H. (2009). *J. Mol. Struct* **932**, 84–89.

[bb8] Choi, J.-H. & Moon, D. (2014). *J. Mol. Struct.* **1059**, 325–331.

[bb9] Choi, J.-H., Oh, I. G., Suzuki, T. & Kaizaki, S. (2004). *J. Mol. Struct* **694**, 39–44.

[bb10] De, G., Szuki, M. & Uehara, A. (1987). *Bull. Chem. Soc. Jpn*, **60**, 2871-2874.

[bb11] Groom, C. R. & Allen, F. H. (2014). *Angew. Chem. Int. Ed.* **35**, 3103-3111.

[bb12] House, D. A. (1973). *J. Inorg. Nucl. Chem.* **53**, 662–671.

[bb13] Moon, D. & Choi, J.-H. (2015). *Acta Cryst.* E**71**, 100–103. 10.1107/S2056989014027479 PMC4331884 25705463

[bb14] Moon, D., Choi, J.-H., Ryoo, K. S. & Hong, Y. P. (2013). *Acta Cryst.* E**69**, m376–m377. 10.1107/S1600536813015456 PMC3772415 24046558

[bb15] Moon, D., Ryoo, K. S. & Choi, J.-H. (2015). *Acta Cryst.* E**71**, 540–543. 10.1107/S205698901500746X PMC4420107 25995875

[bb16] Ooi, S., Komiyama, Y. & Kuroya, H. (1960). *Bull. Chem. Soc. Jpn*, **33**, 354–357.

[bb17] Otwinowski, Z. & Minor, W. (1997). *Methods in Enzymology*, Vol. 276, *Macromolecular Crystallography, Part A*, edited by C. W. Carter Jr & R. M. Sweet, pp. 307–326. New York: Academic Press.

[bb18] Putz, H. & Brandenburg, K. (2014). *DIAMOND*. Crystal Impact GbR, Bonn, Germany.

[bb19] Sandrini, D., Gandolfi, M. T., Moggi, L. & Balzani, V. (1978). *J. Am. Chem. Soc* **100**, 1463–1468.

[bb20] Sheldrick, G. M. (2015*a*). *Acta Cryst.* A**71**, 3–8.

[bb21] Sheldrick, G. M. (2015*b*). *Acta Cryst.* C**71**, 3–8.

[bb22] Westrip, S. P. (2010). *J. Appl. Cryst.* **43**, 920–925.

